# Quantum-to-classical modeling of monolayer Ge_2_Se_2_ and its application in photovoltaic devices

**DOI:** 10.3762/bjnano.15.94

**Published:** 2024-09-11

**Authors:** Anup Shrivastava, Shivani Saini, Dolly Kumari, Sanjai Singh, Jost Adam

**Affiliations:** 1 Computational Materials and Photonics (CMP), Department of Electrical Engineering and Computer Science, University of Kassel, Kassel, Germany; 2 Computational Nano-Material Research Lab (CNMRL), Indian Institute of Information Technology, Allahabad, Uttar Pradesh, India; 3 Department of Electrical Engineering, Indian Institute of Technology, Patna, India; 4 Center for Interdisciplinary Nanostructure Science and Technology (CINSaT), University of Kassel, Kassel, Germany

**Keywords:** 2D materials, density functional theory, hole transport layer, optical properties, solar cells

## Abstract

Since the discovery of graphene in 2004, the unique properties of two-dimensional materials have sparked intense research interest regarding their use as alternative materials in various photonic applications. Transition metal dichalcogenide monolayers have been proposed as transport layers in photovoltaic cells, but the promising characteristics of group IV–VI dichalcogenides are yet to be thoroughly investigated. This manuscript reports on monolayer Ge_2_Se_2_ (a group IV–VI dichalcogenide), its optoelectronic behavior, and its potential application in photovoltaics. When employed as a hole transport layer, the material fosters an astonishing device performance. We use ab initio modeling for the material prediction, while classical drift–diffusion drives the device simulations. Hybrid functionals calculate electronic and optical properties to maintain high accuracy. The structural stability has been verified using phonon spectra. The *E*–*k* dispersion reveals the investigated material’s key electronic properties. The calculations reveal a direct bandgap of 1.12 eV for monolayer Ge_2_Se_2_. We further extract critical optical parameters using the Kubo–Greenwood formalism and Kramers–Kronig relations. A significantly large absorption coefficient and a high dielectric constant inspired the design of a monolayer Ge_2_Se_2_-based solar cell, exhibiting a high open circuit voltage of *V*_oc_ = 1.11 V, a fill factor of 87.66%, and more than 28% power conversion efficiency at room temperature. Our findings advocate monolayer Ge_2_Se_2_ for various optoelectronic devices, including next-generation solar cells. The hybrid quantum-to-macroscopic methodology presented here applies to broader classes of 2D and 3D materials and structures, showing a path to the computational design of future photovoltaic materials.

## Introduction

Reducing fossil fuels and their harmful environmental impact requires improvements in green, sustainable energy sources. Among the various sources of green energy generation, solar energy has been identified as the most promising and expedient because it has the potential to address the current energy demand without making a hazardous impact on the environment [1–2]. Henceforth, researchers have made continuous efforts to design efficient and robust PV devices and solar cells. The systematic study of various solar cells in the last few decades has led to many successful breakthroughs in terms of the stability, efficiency, and cost of PV technology. In the past few decades, perovskite solar cells (PSCs) have emerged as a groundbreaking technology in the field of renewable energy because of their remarkable efficiency and relatively low manufacturing cost [3–4]. These solar cells are based on perovskite-structured compounds, which have demonstrated excellent light absorption, charge-carrier mobilities, and tunable bandgaps [5].

Despite the rapid advancements in PSC technology, some critical issues, such as long-term instability, poor device scalability, and the use of toxic compounds, have yet to be resolved [6]. Recent advancements have seen the integration of two-dimensional (2D) materials with PSCs, opening new avenues for enhancing their performance and addressing current challenges with the PSCs [7–8]. Integrating 2D materials in PSCs can improve their performance. The 2D materials can provide protective layers that work like a shield to the perovskite materials to protect them from environmental degradation caused by moisture and oxygen and provide better device scalability to the PSCs, which makes large-scale production more feasible [9]. Also, the mechanical robustness and flexibility of the 2D materials will give extra room to design the PSCs for a wide range of applications areas [10]. The high carrier mobility and enhanced optoelectronic characteristics can improve the device performance of conventional PSCs [11], and the use of non-toxic 2D materials can improve the performance of PSCs without health risks.

Many researchers recently proposed an ultrathin transport layer-based solar cell as the most expedient photo device for sunlight harvesting. The general scheme of a solar cell comprises various layers including the active/absorber layers, the electron transport layer (ETL) and the hole transport layer (HTL) [12–13]. Both HTL and ETL play a crucial role in achieving a high performance of PV devices. The most common HTL material is spiro-OMeTAD, but it is very expensive [14]. Furthermore, a large number of materials have been reported as ETLs/HTL materials, such as carbon [15], copper iodide [16], CuSbS_2_, NiO, CuSCN [17], ZnO, and CdS [18]. These materials promote efficient extraction of charge carriers and suppress recombination between the perovskite film and the electrodes. But in all these cases, the transport layer thicknesses are taken as sufficiently high. During the last few decades, researchers claimed that ETLs/HTLs of two-dimensional materials could achieve high power conversion efficiency (PCE) and optimum device performance [18–19]. The key features of the 2D materials, rendering them viable for PV applications were discussed in [20].

Because of the advantages of 2D materials, tremendous efforts have been made to invent highly stable and efficient solar cells based on 2D materials. A number of remarkable works have been recently reported using numerous 2D materials, including MXene’s, transition-metal dichalcogenides (TMDCs) and van der Waals structures [21]. Among the various sets of 2D materials, TMDCs (with the general formula of MX_2_, where M is a transition metal and X is a chalcogen) attract the broad attention of the research community regarding their extensive applications in photodevices. This is mainly because of the exceptional optoelectronic behavior of the TMDCs, especially the layer-dependent bandgap and the high absorption of incident sunlight [22–25]. Motivated by this, various TMDCs, including MoS_2_, WS_2_, WSe_2_, and TiS_2_, have been used in PSCs, either as HTL or as ETL. Yin et al. [26] reported a PCE of approximately 17.3% with TiS_2_ nanosheets suspended in isopropanol alcohol in a PSC. 2D sheets of SnS_2_ have been proposed by Zhao et al. [27] as a prospective ETL for PSCs. It achieved a maximum PCE of around 20%. Similarly, other PSCs using different 2D layers of TMDCs have been reported (summarized below in Table 3). Despite the pervasive use of TMDC-based 2D materials in solar cells, their device performance still leaves room for improvement.

Recently, a new class of dichalcogenides (group IV–VI metal chalcogenides) has been successfully synthesized [28–31]. Its remarkable features, such as low cost, abundance, environmental friendliness, and many interesting physical properties, indicate a broad range of possible applications in sustainable electronics and photonics [32]. Notably, a high absorption coefficient has been reported in [33] for an SnS monolayer across the direct absorption edge at 1.3–1.5 eV, rendering it potentially applicable in solar cells and photodetectors. Similarly, Huang et al. [34] showed that a band transition (from indirect to direct) occurs in SnSe_2_ when moving from bulk to monolayers, elucidating its application potential in optoelectronic devices. More group IV–VI dichalcogenides have been discussed in [35–40] for different applications. Although some inceptive work has been done using monolayers of metal dichalcogenides, there are still plenty of opportunities to explore. In line with the same, we have studied the thermoelectric behavior of monolayer Ge_2_Se_2_ in our previous work [41].

**Figure 1:**
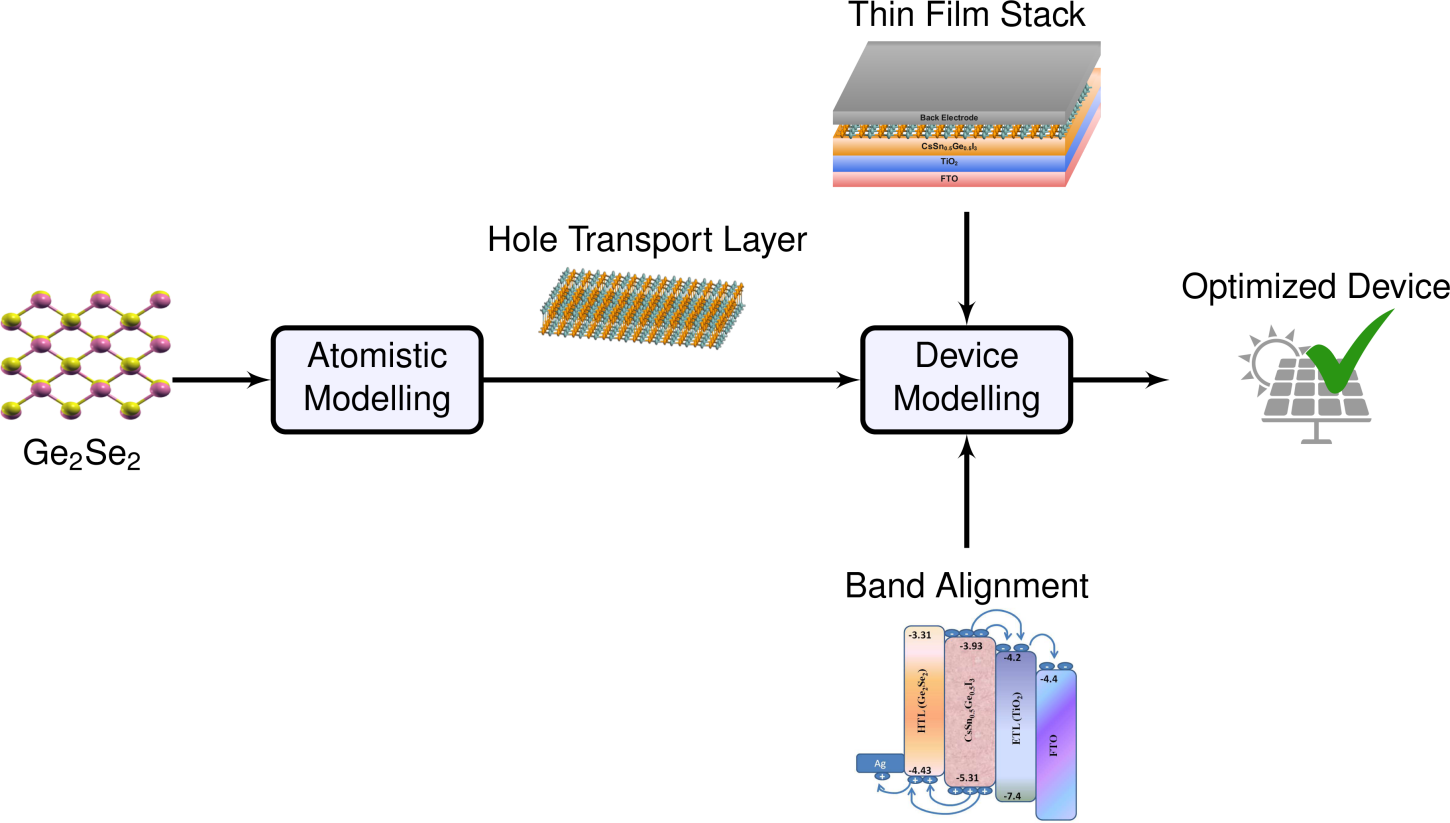
Optoelectronic property investigation of a group IV–VI dichalcogenide monolayer (Ge_2_Se_2_), and its application potential for hole transport layers in Ge_2_Se_2_/CsSnGeI_3_/TiO_2_-based solar cells. Our computational approach combines density functional theory and atomistic modeling with (macroscopic) device modeling to obtain an otimized device performance.

This article aims to investigate the optoelectronic properties of the group IV–VI dichalcogenide monolayer Ge_2_Se_2_ using density functional theory (DFT) and to demonstrate its potential application in photovoltaic solar cells. The proposed Ge_2_Se_2_ monolayer exhibited excellent thermodynamic stability, higher carrier mobilities (due to the presence of valleys in the CB/VB), and good optical response (an interband transition in the visible region). Considering the proper design criteria and selecting an appropriate material for absorbers/ETL, we have designed and modeled the solar cell in a FTO–TiO_2_–CsSn_0.5_Ge_0.5_I_3_–Ge_2_Se_2_–Ag configuration, which outperforms, in terms of their key parameters, other reported works in the literature. The proposed solar cell configuration is environmentally friendly (absence of toxic materials), of low cost, and highly efficient. The computational methodology followed here is depicted in Figure 1, and we provide detailed corresponding instructions and references in the Methods section.

## Results and Discussion

The computational approach can be better explained by separating it into two subsections, namely (a) material simulation using DFT and (b) device simulation using a macroscopic approach, which will be discussed in section Computational Methods.

### Structural and stability analysis

**Figure 2:**
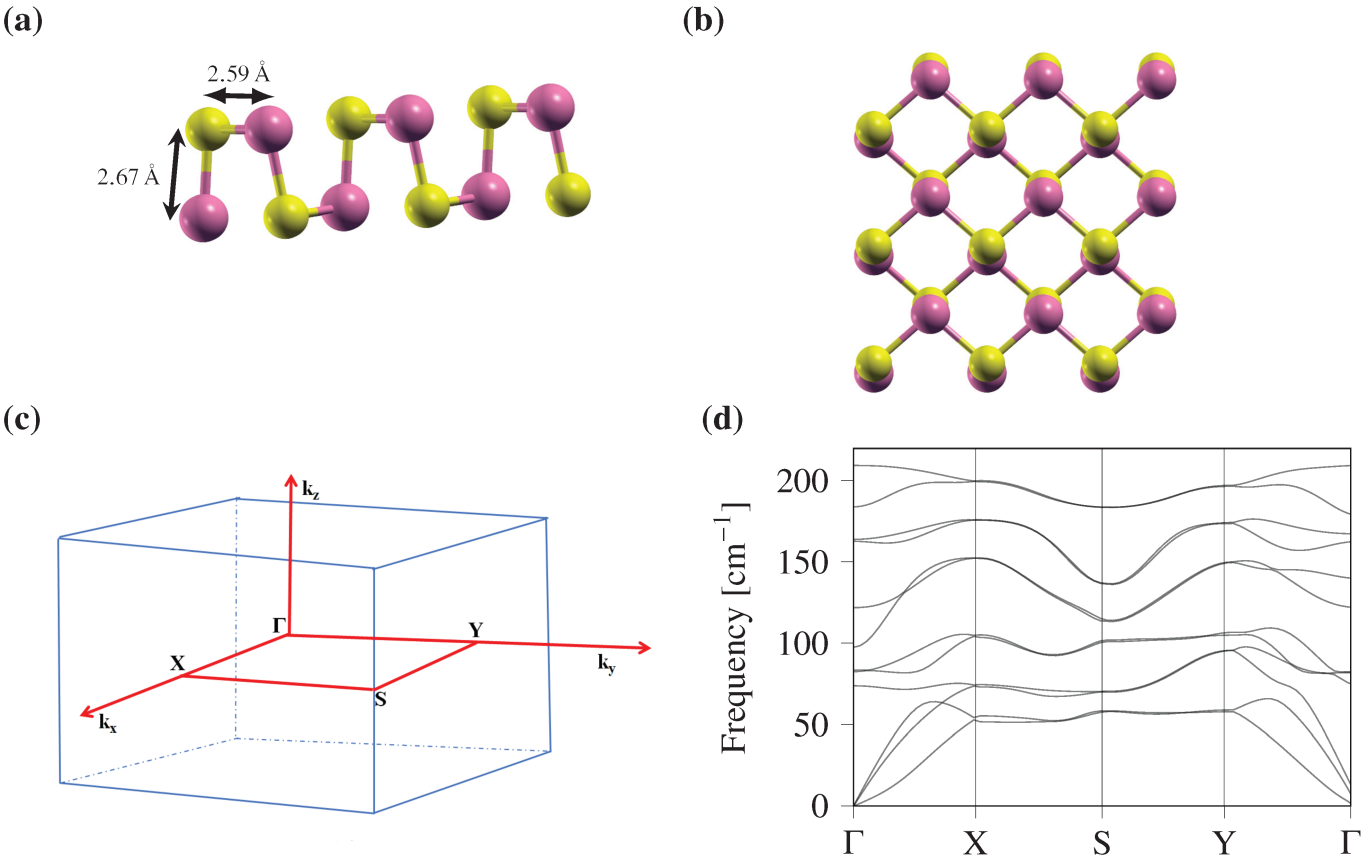
Geometry and stability of Ge_2_Se_2_ monolayers. (a) Side view with the corresponding structural parameters and (b) top view. The exhibited structure is a simple orthorhombic structure with space group symmetry of *Pmn*2_1_ (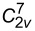) in 2D space. (c) Brillouin zone, where the Γ-X-S-Y-Γ path is used to plot the phonon spectra, and (d) phonon dispersion, confirming the structure’s dynamical stability (Figure 2a,b were redrawn from [41], Figure 2d was re-plotted from [41]).

The schematic atomistic model for monolayer Ge_2_Se_2_ is shown in Figure 2. For better understanding, a side view of monolayer Ge_2_Se_2_ with the corresponding lattice parameters is given in Figure 2a, and the top view of the supercell of the order 4 × 4 is shown in Figure 2b. From Figure 2a, we can observe that the unit cell of monolayer Ge_2_Se_2_ consists of four atoms (two for each Ge and Se). More precisely, we can observe two sub-layers corresponding to a crystallogen (Ge) and a chalcogen (Se), which are designate as top and bottom moieties. From Figure 2, we can also notice that the structure of monolayer Ge_2_Se_2_ is identical to the puckered phosphorene (simple orthorhombic structure) with space group symmetry of *Pmn*2_1_ (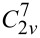) in 2D space.

The geometry-optimized lattice parameters *a* and *b* are found to be 4.13 and 3.99 Å, respectively. The bond lengths between consecutive crystallogen and chalcogen (Ge–Se) are 2.59 and 2.67 Å in the horizontal and vertical directions, respectively. All optimized structural parameters excellently match with previously reported values [41–42]. In addition to the lattice parameters for the monolayer Ge_2_Se_2_, we have optimized the vacuum slab in a normal direction to avoid interlayer interactions. The optimized vacuum level was found to be 23 Å. Furthermore, to test the stability of the crystal structure, we have computed the phonon band dispersion for monolayer Ge_2_Se_2_ within the first Brillouin zone (Figure 2c). The calculated phonon spectra along the high symmetry path Γ-X-S-Y-Γ in the first Brillouin zone are shown in Figure 2d.

The phonon spectrum for monolayer Ge_2_Se_2_ has twelve vibrational modes, of which three are acoustical (low-frequency) modes (transverse acoustic, longitudinal acoustic, and flexural acoustic). The remaining nine (high-frequency) modes correspond to optical modes. The flexural acoustic mode is an out-of-plane transverse acoustic mode, similar to those in other two-dimensional materials such as graphene, phosphorene, and stanene, demonstrating a quadratic nature near the Γ point [43–45]. Figure 2d clearly depicts that all phonon bands throughout the spectrum have non-imaginary frequency, further confirming the investigated structure’s dynamic stability.

### Electronic and transport properties

**Figure 3:**
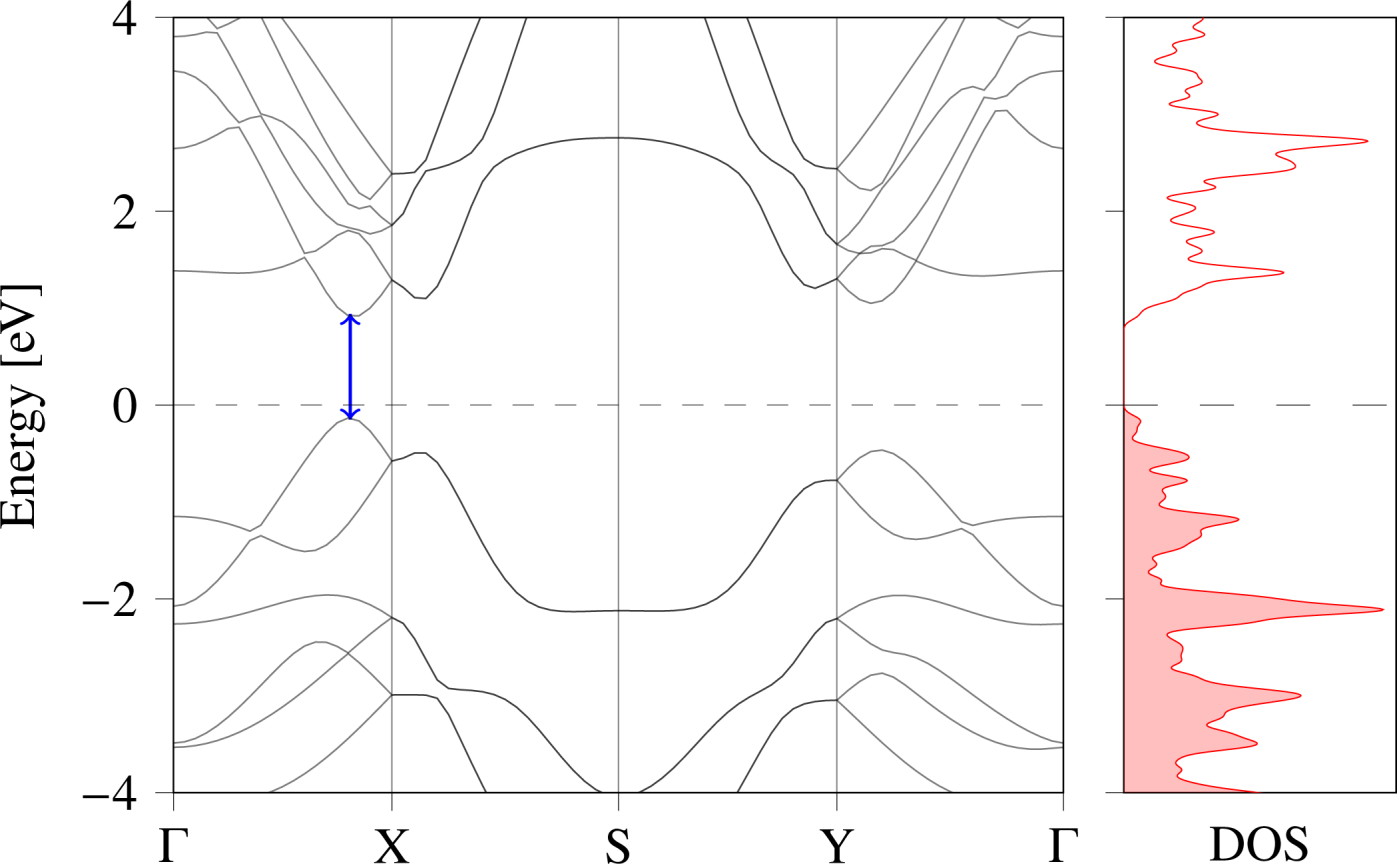
The electronic band structure of monolayer Ge_2_Se_2_, revealing a direct bandgap of 1.12 eV, and the corresponding density of states (The electronic band structure was re-plotted from [41]).

The electronic properties of materials play a crucial role in predicting the material behavior and transport parameters and, hence, its prospective applications. We investigated the electronic properties of monolayer Ge_2_Se_2_ using its electronic band structure and density of states, as shown in Figure 3. To maintain a high degree of accuracy, we calculated the band structure using the HSE06 functional, and the band dispersion along the high symmetry paths Γ-X-S-Y-Γ in the first Brillouin zone, as shown in Figure 3.

Figure 3 depicts that the monolayer Ge_2_Se_2_ is a direct-bandgap semiconductor with a bandgap of the order of 1.12 eV. Valence band maximum (VBM) and conduction band minimum (CBM) are located along the Γ-X path. The computed bandgap value and its dispersion nature are consistent with earlier reported works [41–42,46–47]. The optimum bandgap value and its CBM/VBM positions make it suitable for photovoltaic applications and provide a guideline for selecting the absorber layers in the solar cells. Notably, multivalley and flat bands are present in the energy band structure. The formation of multiple valleys provides extra carrier pockets for transportation, further increasing carrier mobility. Also, the larger valley degeneracy will increase the density of states (DOS) effective mass without influencing the carrier mobility. We have also calculated the total DOS to understand and justify the band structure calculation. Figure 3 shows that the calculated DOS is consistent with the electronic band structure and the bandgap values, further validating the results.

Other properties that will be useful in the simulation of solar cells, such as the effective mass of charge carriers (electrons/holes), conduction/valence band density of states, electron/hole mobility, electron affinity, and work function can be derived from the initial band energy calculation.

We calculated the effective masses of electrons and holes as 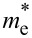 = 0.167*m*_e_ and 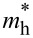 = 0.1768*m*_e_, respectively, which are very close to the values (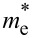 = 0.17*m*_e_, and 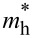 = 0.17328*m*_e_) reported in the literature [46–47]. With that, we estimated the effective DOS values in the CB and the VB to be 0.1732 × 10^19^ /cm^3^ and 0.1887 × 10^19^ /cm^3^, respectively. The electron and hole thermal velocities for monolayer Ge_2_Se_2_ were estimated as 9.43 × 10^5^ m/s and 2.668 × 10^5^ m/s, respectively. The predicted charge carrier mobilities were found to be 187.003 cm^2^/(V·s) and 300.451 cm^2^/(V·s) for holes and electrons, respectively. The high carrier mobility further suggests a potential application as a transport layer in solar cells as well as in the channel materials of FETs. It is important to note that although in the case of solar cells, the transmission of charge carriers takes place in the out-of-plane direction, which is usually considered to be low compared to the in-plane mobility (especially in the bulk materials), the charge carriers still move quickly across the thin HTL because of the ultrathin layer and the strong electric field gradient from the active layer to the electrode. Finally, higher in-plane mobility of the monolayers can compensate the potential limitations of out-of-pane mobility for its prospective uses in solar cells, V-FETs, TFETs, and memristors [48].

**Figure 4:**
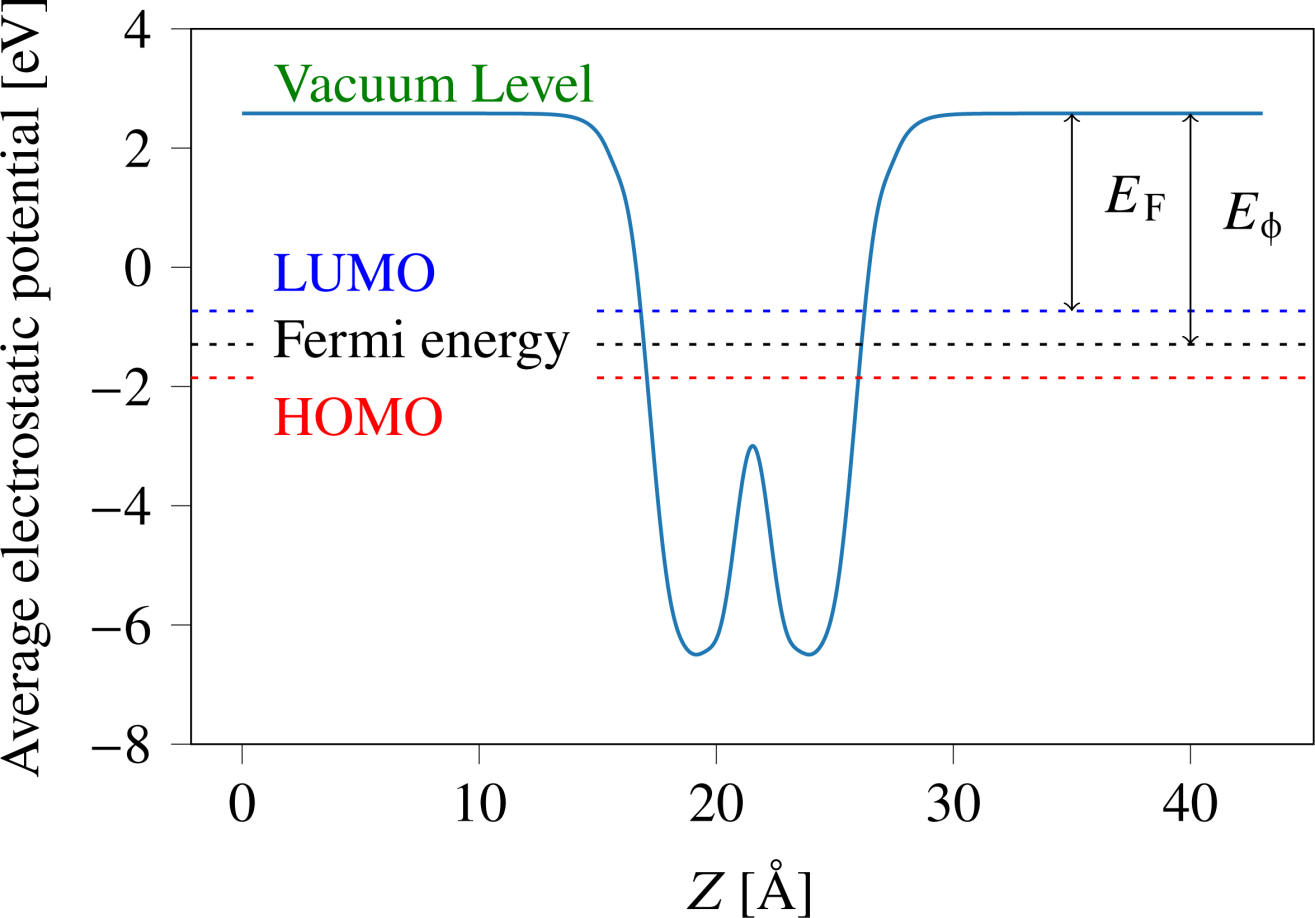
Macroscopic potential distribution vs lattice vector in the normal direction for monolayer Ge_2_Se_2_. In a periodic system, the electron affinity and work function is defined as EA = *E*_Vac_ − *E*_LUMO_, and *E*_ϕ_ = *E*_Vac_ − *E*_F_, where *E*_F_ is the Fermi energy level, *E*_ϕ_ is the work function, EA is the electron affinity, *E*_Vac_ is the vacuum energy level, and *E*_LUMO_ is the LUMO energy level, respectively. The calculated work function and electron affinity are about 3.313 and 3.873 eV, respectively.

In the present calculation, we have calculated the average planar electrostatic potential along the plane as demonstrated in Figure 4. The figure depicts the vacuum energy level and the positions of HOMO and LUMO. The calculated work function and electron affinity for monolayer Ge_2_Se_2_ are about 3.313 and 3.873 eV, respectively, which are comparable with previously reported values of 3.39 eV and 3.9 eV [46–47].

A high degree of agreement for all estimated electronic parameters with earlier reported values validates the accuracy of our computation.

### Optical properties

**Figure 5:**
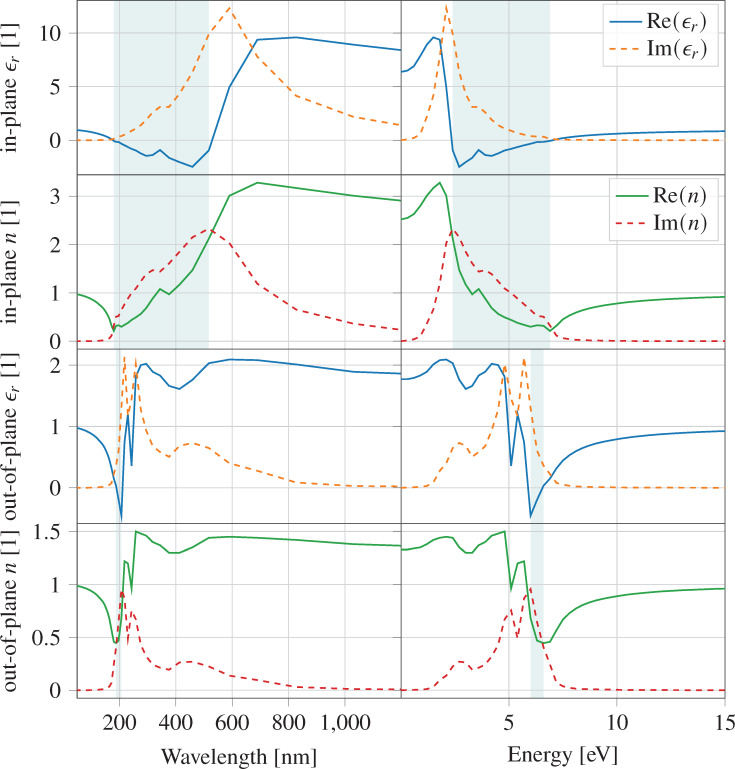
Optical responses of monolayer Ge_2_Se_2_; real (solid) and imaginary (dashed) part of the dielectric constant and refractive index vs wavelength (left) and photon energy (right), for in-plane and out-of-plane excitation relative to the sheet. The teal-colored areas mark the regions of potential metallic behavior (Re(ε*_r_*) *<* 0 or, equivalently, Re(*n*) *<* Im(*n*)), occurring between 180 and 517 nm (2.4 and 6.9 eV) for in-plane, and between 188 and 207 nm (6.0 and 6.6 eV) for out-of-plane excitation.

As discussed in the previous section, monolayer Ge_2_Se_2_ has structural stability and exhibits an optimal electronic behavior with a direct bandgap of 1.12 eV. The DFT results indicate potential applications of the investigated structure in optoelectronic devices. The optical responses at varying photonic energies and the corresponding optical wavelength are summarized in Figure 5, depicting the dielectric function ε*_r_* (real and imaginary parts) and the refractive index *n* for monolayer Ge_2_Se_2_. The real and imaginary components of the dielectric function correspond to dispersive and absorption effects of the material. We observe that the investigated structure exhibits strongly anisotropic optical properties for which the parametric values depend on the direction of the chosen plane.

#### Dielectric function and absorption coefficient

The static dielectric constants (real part of ε*_r_* at ω = 0), along the in-plane (XX) and out-of-plane (ZZ) direction were calculated as 6.37 and 2.4, respectively, suggesting that monolayer Ge_2_Se_2_ is highly polarized along the in-plane direction.

The imaginary part of the dielectric function shows peaks between 2 and 3 eV, along the XX direction, which indicate an interband transition in the visible region (Figure 5). Interestingly, we can observe tracking bands (the region where the energy difference between the conduction and valence bands is approximately constant [49]) around the high-symmetry point S and between X and Y in the first Brillouin zone. Around these points, we notice a small gradient and, thus, large DOS, which further leads to higher transition possibilities.

The absorption coefficient elucidates the rate at which light intensity diminishes while entering the material. It primarily depends upon the imaginary part of the refractive index *k*(λ) = Im{*n*(λ)} and the wavelength of the incident light λ, as per α_abs_ = 4π*k*(λ)/λ. The highest peak of the absorption coefficient lies in the visible region, which we attribute to the direct transition from VBM to CBM. The other peaks correspond to the transition from the top valence band to the conduction band in a band region of the Brillouin zone around the S point. The maximum absorption coefficient is 5 × 10^5^ cm^−1^ along the XX direction. The highest peak for the ZZ direction is calculated as 5.8 × 10^5^ cm^−1^, corresponding to the UV region. We attribute this maximum to the transition from VBM to CBM, while the other peaks designate a transition from VBM to CBM around a wide range of spectra at the S point.

#### Refractive index and extinction coefficient

The real part of the material’s refractive index defines the angle of refraction that the light undergoes while entering a medium. Higher refractive indices refer to lower refraction angles measured from the interface normal. The static refractive indices for monolayer Ge_2_Se_2_ in the direction parallel to the plane (XX) and normal to the plane (ZZ) are approximately 2.5 and 1.4, respectively, as shown in Figure 5. These refractive indices could enable the use of the investigated material as an inner layer coating between the absorber and substrate/electrodes in the design of solar cells.

## Device Modeling

**Figure 6:**
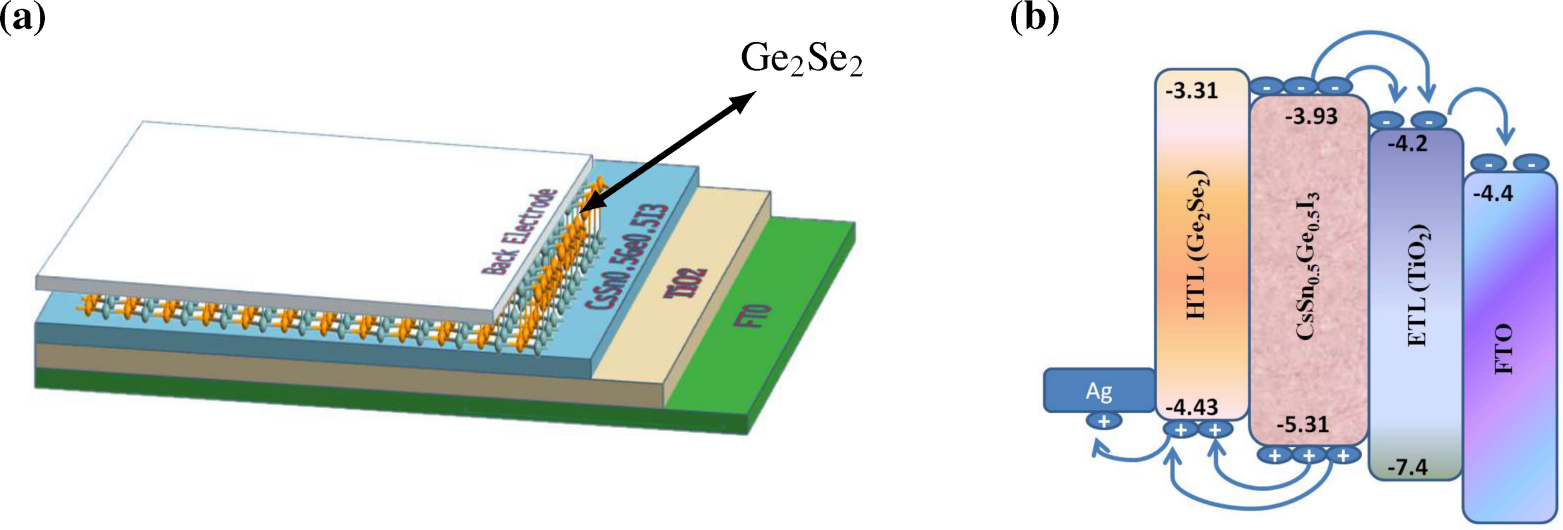
Schematic of the proposed PSC using Ge_2_Se_2_ as HTL; (a) device setup consisting of stacked layers of FTO–TiO_2_–CsSn_0.5_Ge_0.5_I_3_–Ge_2_Se_2_–Ag; (b) band offset among different PSC layers, demonstrating the ease of charge-transfer from the active layers to the respective transport layers.

The high carrier mobility, optimum bandgap, and suitable optical characteristics of monolayer Ge_2_Se_2_ as discussed in the previous section, further motivated us to design a photovoltaic solar cell using monolayered Ge_2_Se_2_. The easy fabrication, structural stability, and proper band alignment with the absorber material make it a promising hole transport layer (HTL) in the proposed solar cell. The proposed solar cell is a low-cost, environmentally friendly, scalable Perovskite solar cell (PSC), with Ge_2_Se_2_ as a HTL. The schematic of the proposed solar cell is shown in Figure 6a.

The proposed structure consists of a lead-free Perovskite layer as an absorber, titanium dioxide (TiO_2_) as ETL, and Ge_2_Se_2_ as HTL. A 100 nm thick layer of FTO is used to encapsulate the transport and absorber layers and treated as a conductor in this simulation. Regarding an absorber material corresponding to the proposed HTL, a minimum valence band offset between these two layers has been considered as a necessary condition for achieving optimum performance. In addition to this, environmental friendliness, stability, and cost-effectiveness were also considerations to choose CsSn_0.5_Ge_0.5_I_3_ as an absorber layer. After selecting the absorber layer, the ETL has been chosen based on a matching conduction band offset with the absorber layer and the high electron mobility. Among the various options, TiO_2_ offers minimum band offset and ease of synthesis. The front and back contacts are supposed to be ohmic and are made of silver (Ag) to maintain proper band alignment and reduce the band offset. It has been reported that tuning the CBM and VBM positions to keep a low band offset leads to superior device performance. Figure 6b exhibits the position of CBM/VBM in consecutive layers of the proposed structure. This further demonstrates the ease of charge transfers from the absorber to the respective transport layers.

The thickness and other simulation parameters for the active layer and the ETL were taken from the literature [17,50–51], while all the simulation parameters for Ge_2_Se_2_ have been derived from the DFT calculations as discussed in the previous section. All simulation parameters are summarized in Table 1

**Table 1:** Simulation parameters for the proposed PSC.

Device parameter	FTO [50]	Ge_2_Se_2_ [our calculations]	CsSn_0.5_Ge_0.5_I_3_ [51]	TiO_2_ [17]

Bandgap (*E*_g_) (eV)	3.4	1.12	1.5	3.26
Electron affinity (eV)	4.5	3.31	3.9	4.2
Dielectric constant (ε*_r_*)	9.1	6.37	28	10
Conduction band effective DOS (cm^−3^)	1.1 × 10^19^	0.1732 × 10^19^	1.0 × 10^19^	2.0 × 10^17^
Valence band effective DOS (cm^−3^)	1.1 × 10^19^	0.1887 × 10^19^	1.0 × 10^19^	6.0 × 10^17^
Electron thermal velocity (m·s^−1^)	1 × 10^7^	9.47 × 10^5^	1 × 10^7^	1 × 10^7^
Hole thermal velocity (m·s^−1^)	1 × 10^7^	2.66 × 10^5^	1 × 10^7^	1 × 10^7^
Electron mobility (cm^2^·V^−1^·s^−1^)	20	300.451	974	100
Hole mobility (cm^2^·V^−1^·s^−1^)	10	187.003	213	25

### Performance estimation

Using the proposed model, we have estimated the performance of the solar cell in terms of open-circuit voltage (*V*_oc_), short-circuit current density (*J*_sc_), fill factor (FF), and power conversion efficiency (η). The performance of solar cells largely depends on various parameters, including the thickness of transport layers, absorber layers, and absolute temperature. Therefore, to get the optimum performance of the proposed device, we have optimized the performance of the cell module by varying the layer thicknesses of HTL, ETL, and absorber. Also, we have tested the performance of the device under the influence of varying defects densities in the active layer and the interfaces. The optimization corresponding to different parameters is summarized in the following sections.

#### Effect of HTL thickness variation

**Figure 7:**
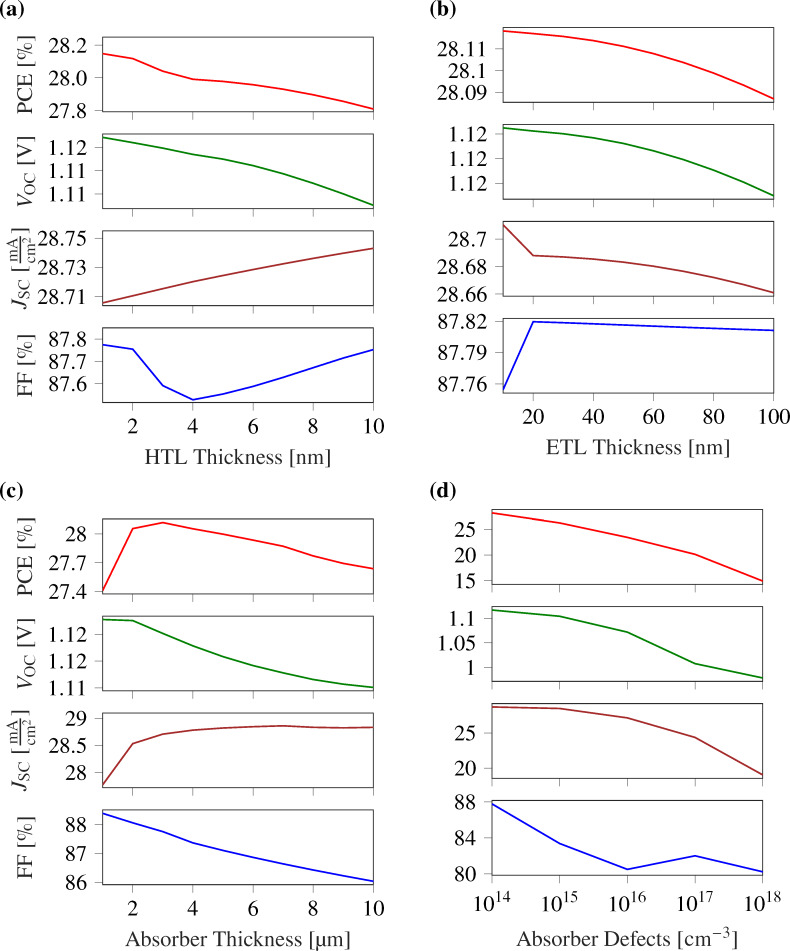
PSC performance parameters as functions of (a) HTL thickness, (b) ETL thickness, (c) absorber thickness, and (d) absorber defects. The device exhibits optimal performance at layer thicknesses of ETL, absorber, and HTL of 20 nm, 2 µm, and 1 nm, respectively.

While the absorber layer is the crucial component of a solar cell, the role of the HTL cannot be overlooked because the choice of HTL plays a significant role in ensuring efficient charge transport, reducing recombination losses, and overall device stability. Therefore, numerous efforts have been made in the past to investigate suitable HTL materials for efficient solar cells [52–54]. The main function of the HTL is to capture the holes generated from the absorber layer and transfer them to the respective electrode. The thickness of the HTL also influences the device performance significantly. To optimize the HTL thickness for optimal device performance, we assumed a layer thickness of monolayer Ge_2_Se_2_, ranging from 1 to 10 nm. Figure 7a depicts the change of the performance parameters upon varying the HTL thickness.

It can be observed from Figure 7a that the device parameters decrease with higher HTL thickness. This behavior is mainly due to the appearance of a higher series resistance at larger HTL thicknesses. Also, a thick HTL may increase the chances of charge carrier recombination. Nevertheless, the performance degradation is very slight because of the ultrathin HTL layer proposed here. It is worth mentioning that there is an incremental rise in the short-circuit current density with thicker HTL. This is due to less carrier losses owing to reduced recombination at the interface of HTL and absorber layer and better surface cleavage because of the thick HTL [55–56]. Figure 7a indicates that 1 nm of HTL yields superior performance in terms of PCE, *V*_oc_, *J*_sc_, and FF with values of 28.148%, ≈1.11 V, 28.70 mA·cm^−2^, and ≈87.77% FF, respectively.

#### Effect of ETL thickness variation

In conjunction with the HTL, the ETL is responsible for collecting electrons generated in the active absorber layer. It is also necessary to optimize the ETL thickness to get an optimal device behavior. The change of the device parameters with varying ETL thickness is shown in Figure 7b. A higher thickness of the ETL leads to a worse device performance. When the layer thickness increases, the series resistance in the cell will also increase, and the light transmittance through the ETL decreases. The latter affects photogeneration in the perovskite layer, reducing *J*_sc_ and, consequently, the cell efficiency. Also, one can notice that the fill factor initially increases and then decreases mainly due to the small reverse saturation current for lower values of the ETL thickness. Later on, at higher ETL thicknesses, the fill factor decreases with increase in series resistance, and the cell efficiency decreases [57]. Since the ETL thickness is low, the impact on the performance degradation is insignificant. After examining all parameters, we found an optimized ETL thickness of 20 nm in our proposed device. The optimized values of the solar cell parameters at the optimal ETL thickness are 28.11%, ≈1.11 V, 28.68 mA·cm^−2^, and ≈87.81% for PCE, *V*_oc_, *J*_sc_, and FF, respectively.

#### Effect of absorber thickness variation

The absorber layer thickness is the most important and critical device parameter. The role of the absorber layer is to absorb the photons and generate charge carriers required for the current flow. It is intuitive to have a thick absorber layer for a higher current density. However, because the absorber thickness further affects the recombination rate, optimizing it for optimal device performance is necessary. The impact of varying absorber thickness on the different device parameters is summarized in Figure 7c. The absorber layer thickness was varied from 1 to 10 µm to obtain the optimal performance of the proposed device. Figure 7c shows that the short-circuit current density (*J*_sc_) increases with higher absorber thickness up to a thickness of 4 µm, before reaching a constant value. The maximum short-circuit current density (*J*_sc_) was calculated as 28.86 mA·cm^−2^ corresponding to an absorber thickness of 4 µm. The current variation followed the Beer–Lambert law, which justifies that more absorption will occur when the thickness of the absorber layer increases. A thicker absorber layer allows more photons to get absorbed, which leads to the generation of more electron–hole pairs. These generated electron–hole pairs, collected by the respective transport layers, contribute to the higher current density [58].

It is worth noticing that there is a significant impact of the absorber thickness on almost all parameters. The PCE increases from 27.4% to 28.11%, and the short-circuit current *J*_sc_ value reaches up to 28.86 mA·cm^−2^. We observed that there is a significant reduction in FF and *V*_oc_ at higher absorber thicknesses, primarily because of the increasing series resistance and higher losses [59]. We set the optimal absorber thickness at 2 µm, above which the parameter variation was only very little.

#### Effect of defects in the absorber layer

Halide pervoskites are generally considered as defect-resilient; yet, deep level defects shows significant impact on the device performance [60–61]. Therefore, it is important to analyze their impact on the cell performance for better device design. Figure 7d shows the variation of the cell parameters at varying absorber defect densities from 10^14^ cm^−3^ to 10^18^ cm^−3^. Figure 7d shows that higher defect densities lead to worse device performance. This is due to the decrement in the lifetime of the charge carriers because of the shorter diffusion length, which results from the higher defect densities [62]. We can observed that the performance parameters decrease significantly above defect densities of 10^15^ cm^−3^.

#### Effect of interface defects between active and transport layers

**Figure 8:**
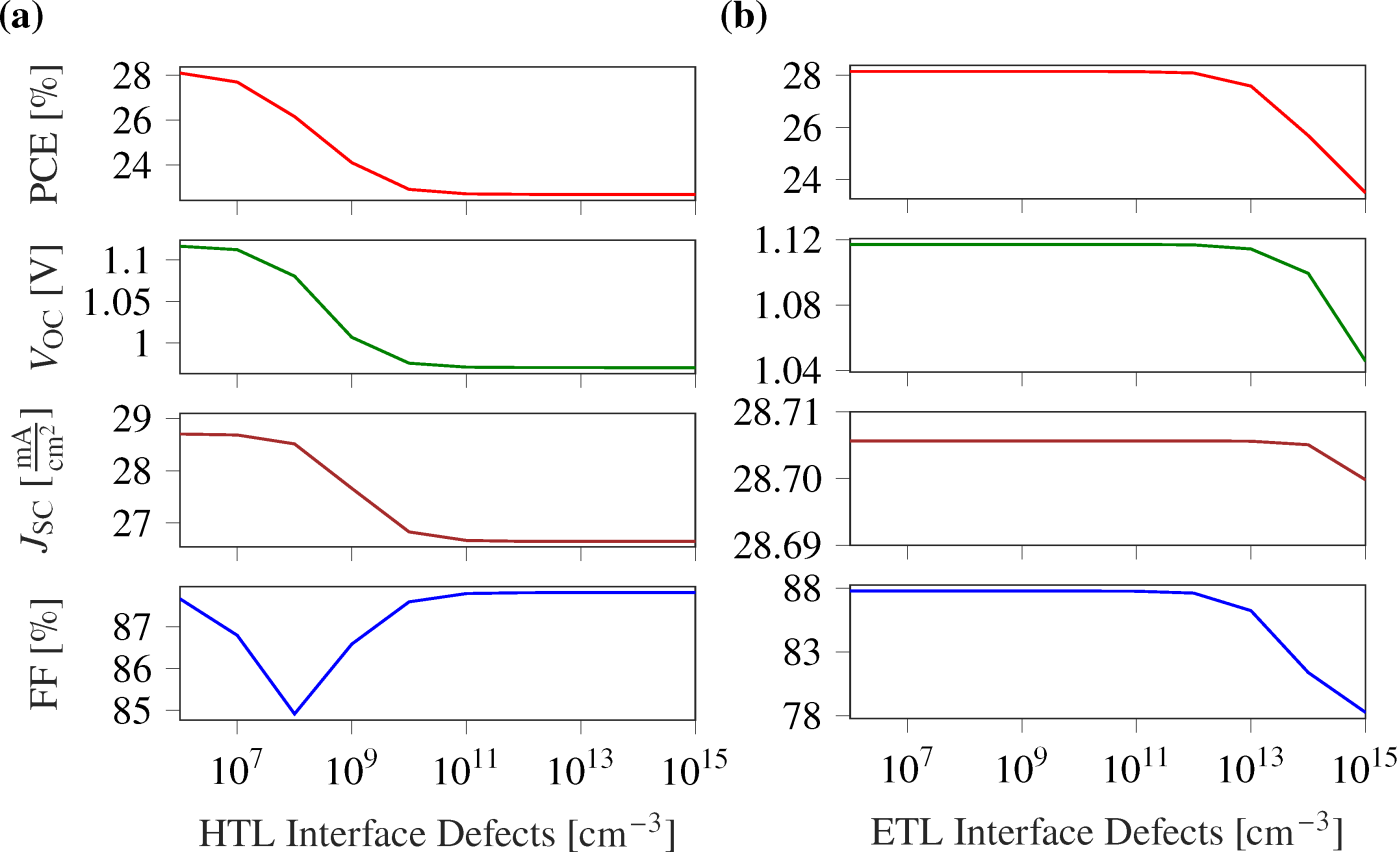
Variation of PSC performance parameters with interface defects between (a) ETL and active layer, and (b) active layer and HTL. Because of the ultrathin HTL layer, the interface at the absorber/HTL is more sensitive, and the device performance degrades for interface defect densities above 10^7^ cm^−3^. However, the ETL/absorber interface exhibits an excellent performance up to the interface defects of 10^12^ cm^−3^, after which the device performance starts to deteriorate.

The photovoltaic behavior of halide perovskites is significantly affected by the properties of the interfaces. This is because a majority of defects arises at the junction between two layers during the fabrication of the device, caused by mismatches in the lattice structures, which lead to dangling bonds. These defects at the junctions play a critical role in the performance of the device as they contribute to increased local recombination at the interface. Within this particular PSC, two critical interface layers exist, namely, the interface between the absorber and the ETL and the interface between the absorber and the HTL. We analyzed the device performance by varying the defect densities at both interfaces within the range of 10^6^ cm^−3^ to 10^15^ cm^−3^. Because of the ultrathin HTL layer, the absorber/HTL interface is more sensitive, and the device performance is anticipated to degrade above interface defect densities of 10^7^ cm^−3^. However, the ETL/absorber interface exhibits excellent performance up to interface defects of 10^12^ cm^−3^ (Table 2), after which the device performance starts to deteriorate (Figure 8).

**Table 2:** Performance of the proposed PSC under 1.5G solar illumination.

Device parameter	Value

Open circuit voltage (*V*_oc_)	1.11 V
Short circuit current density (*J*_sc_)	28.70 mA·cm^−2^
Fill factor (FF)	87.66%
Power conversion efficiency (PCE)	28.10%

**Figure 9:**
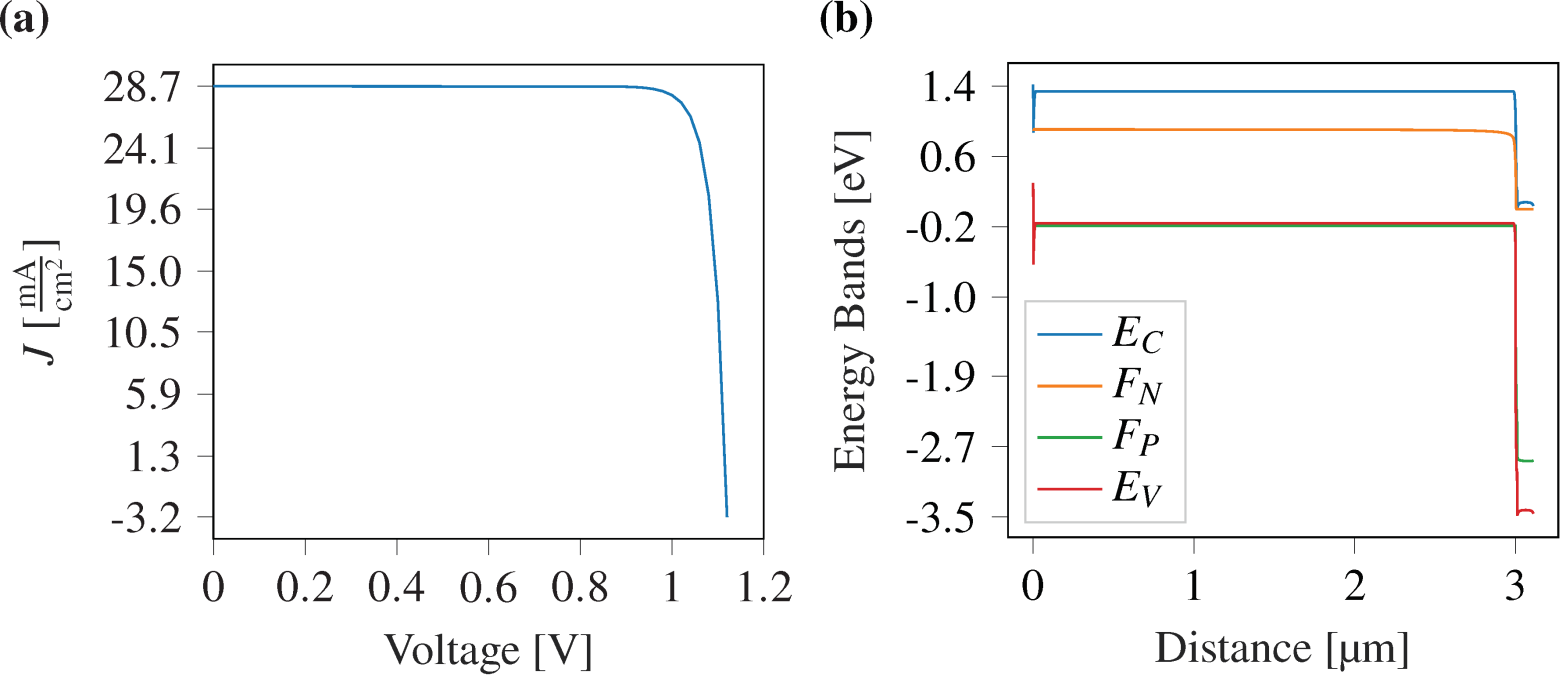
(a) Current density vs voltage curve for the proposed PSC, demonstrating a short-circuit current density and an open-circuit voltage of 28.7 mA·cm^−2^ and 1.11 V, respectively. (b) Energy band diagram for the proposed solar cell, where *E*_C_ stands for conduction band and *E*_V_ stands for valence band. *F*_N_ and *F*_P_ stand for quasi-Fermi levels of electrons and holes, respectively, at *T* = 300 K.

The computed current density vs voltage curve is shown in Figure 9a, and the energy band diagram corresponding to the different layers is demonstrated in Figure 9b. We have also compared the proposed device with the combination of different transport layers in Table 3. It demonstrates that the proposed PSC structure excels in terms of performance parameters compared to other reported structures.

**Table 3:** Comparison of the PSC performance with earlier reported works.

Device configuration	*V*_oc_ (V)	*J*_sc_ (mA·cm^−2^)	FF (%)	PCE (%)

Ag/TiO_2_/PSK/MoS_2_/ITO [19]	0.93	26.24	83.0	29.43
Au/Cu_2_O/PSK/TiO_2_/FTO [63]	0.96	15.8	59.0	8.93
Au/MoS_2_/PSK/WS_2_/FTO [64]	0.96	27.3	87.62	22.17
Au/Spiro–OMeTAD/PSK/TiS_2_/FTO [26]	0.95	20.05	66.90	12.75
Ag/Ge_2_Se_2_/PSK/TiO_2_/FTO [This work]	1.11	28.70	87.66	28.10

## Computational Methods

**Figure 10:**
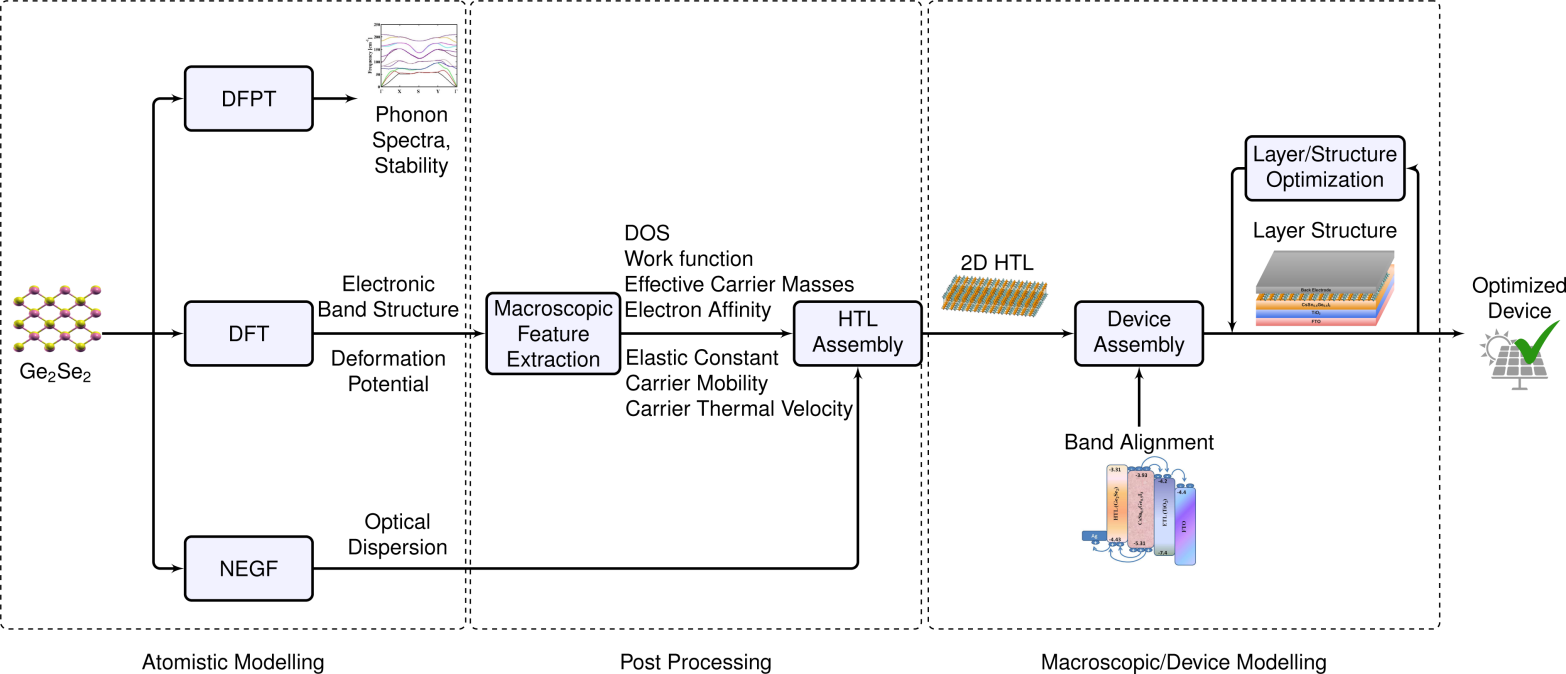
Computational pathway to estimate the materials’ behavior and application in a PSC. First step – DFT geometry optimization, optimization of computational parameters, and electronic band structure and deformation potential extraction. Second step – test of the structural stability via density functional perturbation theory (DFPT): We compute the phonon spectra to verify the structure’s dynamical stability. Third step – DFT-based electronic property estimation: We utilize the HSE functional to predict the band structure. Fourth step – DFT post-processing: (a) Extraction of the device-related parameters such as DOS, charge mobility, effective masses, thermal velocities, electron affinity, and work functions from fundamental physics equations together with DFT-post processing; (b) calculation of the optical behavior based on the non-equilibrium Green’s function (NEGF) and the Kubo–Greenwood approaches. Fifth step – device design, simulation, and optimization: After proper band alignment and suitable material choices for the transport and active layers, we design a stacked solar cell, simulate it (in the SCAPS simulator), and optimize the individual layers for the best device performance.

The methodology presented here (illustrated in Figure 10) is more generally valid and will apply to broader classes of 2D and 3D materials and structures. Therefore, we believe this section might serve as a mini tutorial for the computational design of future photovoltaic materials.

### Material simulation using DFT

The structural and electronic properties have been investigated using an ab initio computational approach within the density functional theory (DFT) framework as implemented in the Quantum Espresso (QE) code [65–66].

We started our calculation to relax the structure at its most stable state (minimum energy) using the Broyden–Fletcher–Goldfarb–Shanno (BFGS) algorithm for a systematic computational study. It has been reported that the hybrid functional is more accurate in predicting the material behavior than local density approximation (LDA) [67] or generalized gradient approximation (GGA) [68] for modeling the exchange–correlation. Hence, for greater accuracy, we have used the Perdew–Burke–Ernzerhof (PBE) [69] and Heyd–Scuseria–Ernzerhof (HSE06) [70] functionals to model the exchange–correlation interactions with a screening parameter of 0.1 and mixing parameters of 25%. An ultrasoft, scalar relativistic pseudopotential of Troullier–Martins type [71] has been used to model the core corrections in the calculations. The valence electronic configuration for Ge and Se is taken as 4*s*^2^4*p*^2^3*d*^10^ and 4*s*^2^4*p*^4^3*d*^10^, respectively, and all electron-like wave functions have been generated using the projected augmented wave (PAW) method. A plane-wave basis set has been used to consolidate the interaction between core and valence electrons with an optimized threshold energy of 60 Ry. For efficient and precise calculations, the Brillouin zone has been sampled into 13 × 13 × 1 K-mesh points (using the Monkhorst–Pack scheme [72]) and high-energy/force convergence criteria of 10^−10^ eV/10^−10^ eV/Å^3^, respectively, have been set for two consecutive self-consistent cycles. A sufficiently high vacuum of 23 Å is applied perpendicularly to avoid interlayer interactions.

The stability of the Ge_2_Se_2_ monolayer was estimated by phonon dispersion. The phonon dispersion has been calculated using density functional perturbation theory (DFPT), as implemented in the QE package. To compute the phonon spectra, we have estimated the second-order interatomic force constant using the relation [73]:


[1] $$\Phi_{\alpha \beta }=\frac{\partial^{2}\phi }{\partial r_{\alpha }\partial r_{\beta }}.$$

To maintain high accuracy, a large plane-wave mesh cutoff of 120 Ry is considered throughout the calculations.

### Extraction of device-relevant parameters

To design a solar cell, we can derive some parameters from the DOS and *E*–*k* dispersion curves, such as the effective masses of electrons (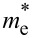) and holes (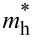), the effective DOS in the conduction and valence bands (*N*_c_ and *N*_v_), electron and hole thermal velocities (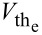 and 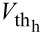), electron and hole mobilities, electron affinity, and work function. We outline an efficient approach to calculating these device parameters briefly below.

According to the *K*–*P* model, the charge carrier effective masses are given as 
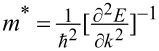
, where ℏ is the reduced Plank constant, *E* is the charge carrier’s energy, and *k* is a wave vector. To estimate the effective masses of electrons and holes, we used a curve-fitting algorithm (weighted least squares method) within a small parabolic section of the conduction band minima (CBM) and valence band maxima (VBM).

Using the charge carriers’ effective masses, the effective DOS in the conduction and valence bands can be estimated as [74–75]:


[2]

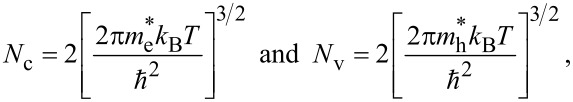



where *N*_c_ and *N*_v_ are the effective DOS in the CB and VB, respectively, and the charge carriers’ effective masses are 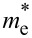 and 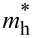 for electrons and holes, respectively. *k*_B_, and *T* denote the Boltzmann constant and the absolute temperature, respectively.

We also calculated the electron and hole thermal velocities using their effective masses by [75–76]:


[3] $$v_{{\text{th}}_{\text{e}}}=\sqrt{\frac{3k_{\text{B}}T}{m_{\text{e}}^{*}}}\text{ and }v_{{\text{th}}_{\text{h}}}=\sqrt{\frac{3k_{\text{B}}T}{m_{\text{h}}^{*}}}.$$

To compute the electron and hole mobilities, we have employed the deformation potential techniques [77] along with the acoustic phonon-limited approach. To estimate the phonon’s limited mobility, we followed Bardeen–Shockley’s approach [78], which says that the atomic displacement associated with a long wavelength acoustic phonon causes crystal deformation and significantly shifts the electronic energy dispersion. This change in the energy band edge with the differential displacement is characterized through the electron–phonon coupling Hamiltonian (*H*_el−ph_) as:


[4]

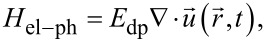



where *E*_dp_ is the deformation potential and 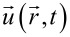 is the displacement at the spatial coordinate 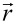 and time *t*. Further, van de Walle [79] suggested that the electron–phonon coupling Hamiltonian (*H*_el−ph_) can be simplified to


[5] $$\partial E\left(k\right)=E_{\text{dp}}\frac{\partial V}{V_{0}},$$

where ∂*E*(*k*) is the induced band edge shift due to the acoustic phonon and ∂*V*/*V*_0_ is the fractional change in the unit cell volume due to the strain implication. After getting the value of the deformation potential using Kawaji theory [80], we can formulate the phonon-limited mobility (μ_2D_) because of the interaction of the charge carriers with low acoustic phonons as [81]:


[6]

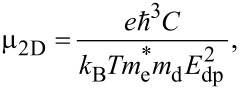



where *C* denotes the elastic constant of a 2D material and 
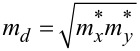
 is the geometric mean of the effective masses. The elastic constant *C* can be derived by knowing the interatomic force constant, calculated applying a uniaxial strain δ in the direction of lattice vector *a*:


[7] $$C=\frac{\partial^{2}E}{\partial^{2}\delta }\frac{1}{A_{0}},$$

where *A*_0_ is the surface area of the unit cell.The deformation potential *E*_dp_ can be calculated using a band edge variation formulation *E*_dp_ = ∂*E*_edge_/∂δ, where *E*_edge_ is the valence and conduction band edge, and *E*_dp_ is computed by imposing a compressive and tensile strain δ to the unit cell. After substituting the values of *E*_dp_, elastic constant *C*, and average effective mass *m*_d_, we calculate the mobility of the charge carriers (for electrons and holes).

To calculate the charge transfer and use it in the solar cell, it is required to know the electron affinity and work function of monolayer Ge_2_Se_2_. The electron affinity is calculated as *E*_A_ = *E*_Vac_ − *E*_LUMO_, where *E*_A_ is the electron affinity, *E*_Vac_ is the vacuum energy level, *E*_LUMO_ is the LUMO energy level. The ionization potential is calculated as IP = *E*_Vac_ − *E*_HOMO_, where IP, *E*_Vac_, and *E*_HOMO_ are ionization potential, vacuum energy level, and HOMO energy level, respectively. The electron affinity at a semiconductor surface is defined as the energy needed to carry an electron from the vacuum to the bottom of the conduction band. Similarly, the work function is defined as the minimum energy required by an electron of the material to escape into the vacuum [82–83]. *E*_A_ can be calculated using DFT as the energy difference between the vacuum level (*E*_Vac_) and the Kohn–Sham (KS) eigenvalues relative to the bottom of the conduction band. Along with the DFT-derived parameters, some excited state eigenfunctions in terms of absorption coefficient and dielectric constant are also required to simulate solar cell-based devices. An optimum way of extracting these optical parameters from ground-state eigenvalues is described below.

### Extraction of optical properties

To explore the optical properties, including dielectric constant, refractive index, absorption constant, extinction coefficient, and susceptibility for monolayer Ge_2_Se_2_, we employed the non-equilibrium Green’s function (NEGF) and the Kubo–Greenwood formalisms [84] as described in the Synopsys-ATK [85]. A double zeta-polarized (DZDP) basis set has been used with a localized pseudo atomic orbital to calculate optical properties and characterize the valence electrons of the constituent atoms. To maintain high accuracy, a large plane-wave mesh cutoff of 120 Ry is considered throughout the calculations. We computed the optical parameters within the range of 0–30 eV. They stem from the dielectric function (using the Kramers–Kronig relations [86]), further estimated through the susceptibility tensors. The susceptibility tensors and complex dielectric functions are related as:


[8]

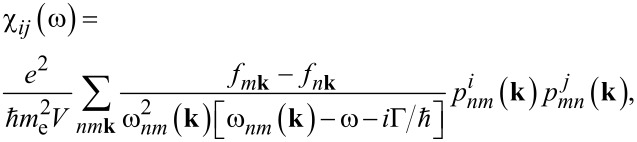



where 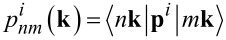 is the *i*-th component (labeling electrons) of the momentum operator between states *n* and *m*, *m*_e_ and *e* are the electron mass and charge, respectively, *V* is the volume, Γ the energy broadening, and ℏω*_nm_* = *E_n_* − *E_m_* and *f_n_***_k_** is the Fermi function (evaluated at the energy *E_n_*(**k**)).

The response coefficients, that is, the relative dielectric permittivity (ε*_r_*), the polarizability (α), and the optical conductivity (σ), are related to the susceptibility as:


[9] $$\varepsilon_{r,ij}\left(\omega \right)=\left(1+\chi_{ij}\left(\omega \right)\right),$$

[10] $$\alpha_{ij}\left(\omega \right)=V\varepsilon_{0}\chi_{ij}\left(\omega \right),$$

[11] $$\sigma_{ij}\left(\omega \right)=-i\omega \varepsilon_{0}\chi_{ij}\left(\omega \right),$$

with *i*,*j* ∈ {1,2,3}. Solving and separating Equation 9 for the imaginary part of dielectric function, we can calculate the refractive index and extinction coefficient as


[12] $$\varepsilon_{2}\left(\omega \right)=\frac{2e^{2}\pi }{\Omega \varepsilon_{0}}\sum_{k,v,c}{\left|\langle \Psi_{k}^{c}|u\cdot r|\Psi_{k}^{v}\rangle \right|}^{2}\delta \left[E_{k}^{c}-E_{k}^{v}-E\right].$$

Here, 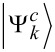 and 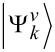 are the conduction and valence band Bloch states with energies 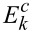 and 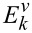, respectively, **r** is the position of the electron, and **u** is the polarization of light. *e* is the electronic charge, and Ω is the volume. Via the well-known relationship between relative permittivity and refractive index 
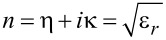
, we finally get the following relationships for the refractive index and the absorption coefficient.

Refractive index/optical density:


[13] $$\eta \left(\omega \right)={\left[\sqrt{\varepsilon_{1}^{2}\left(\omega \right)+\varepsilon_{2}^{2}\left(\omega \right)}+\frac{\varepsilon_{2}\left(\omega \right)}{2}\right]}^{1/2}$$

Extinction coefficient:


[14] $$\kappa \left(\omega \right)={\left[\sqrt{\varepsilon_{1}^{2}\left(\omega \right)+\varepsilon_{2}^{2}\left(\omega \right)}-\frac{\varepsilon_{2}\left(\omega \right)}{2}\right]}^{1/2}$$

Absorption coefficient:


[15] $$\alpha \left(\omega \right)=\sqrt{2\pi }{\left[\sqrt{\varepsilon_{1}^{2}\left(\omega \right)+\varepsilon_{2}^{2}\left(\omega \right)}-\varepsilon_{1}\left(\omega \right)\right]}^{1/2}$$

### Device modeling

To investigate the performance of the proposed solar cell, we performed a numerical simulation using SCAPS-1D, which solves the fundamental semiconductor equations such as drift–diffusion, Poisson’s equation, and continuity equations as:


[16] $$\frac{\partial }{\partial x}\left(\varepsilon_{0}\varepsilon_{r}\frac{\partial \phi }{\partial x}\right)=-q\left(p-n+N_{D}^{+}-N_{A}^{-}+\frac{\rho_{\text{def}}}{q}\right),$$

[17] $$-\frac{\partial J_{n}}{\partial x}-U_{n}+G_{n}=\frac{\partial n}{\partial t},$$

and


[18] $$-\frac{\partial J_{p}}{\partial x}-U_{n}+G_{n}=\frac{\partial n}{\partial t},$$

where 
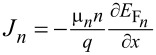
 and 
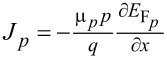
 are the electron and hole current densities at the Fermi levels *E*_F_*_n_* and *E*_F_*_p_*, respectively. ε_0_, ε*_r_*, ϕ, *q*, *n*, and *p* denote the absolute and relative permittivity, the electrostatic potential, the electronic charge, and the electron and hole concentrations, respectively. The charge carrier mobilities, generation, and recombination rates for electrons and holes are represented by μ*_n_*, *G_n_*, *U_n_*, μ*_p_*, *G_p_*, *U_p_*, respectively. All these parameters are functions of the position coordinate *x*.

During the simulation of the proposed device, it is assumed that the reflection of the top surface is zero. In contrast, the reflection from the back surface is considered as 100%, so the all incident photons have greater energy than the bandgap of the materials and can contribute to generating charge carriers. The generated charge carrier mobility of the chosen material assists in delivering it up to the contacts.

## Conclusion

This contribution investigates the electronic and optical properties of monolayer Ge_2_Se_2_ using DFT. The structure of the monolayer is properly optimized and relaxed for its lowest energy state. Tough threshold criteria have been chosen to maintain high precision in the calculation, along with a hybrid functional for modeling the exchange–correlation interactions. The non-imaginary frequencies in the phonon spectra confirm the stable geometry of the investigated structure. The electronic properties of the monolayer have been investigated in detail in terms of energy band diagram, effective masses of the charge carriers, mobility of electrons and holes at room temperature, the total density of states along with effective densities in conduction and valence band, electron affinity, and ionization potentials. In addition, the optical behavior of monolayer Ge_2_Se_2_ has been discussed through exhaustive calculations of the material’s absorption coefficient, dielectric constants, refractive index, and extinction coefficient. The optimum bandgap (1.2 eV), high carrier mobility, and promising optical characteristics of monolayer Ge_2_Se_2_ further led us to investigate the application of the investigated structure. To show the application of monolayer Ge_2_Se_2_, a perovskite solar cell has been proposed using monolayer Ge_2_Se_2_ as HTL. The optimal performance was determined under AM1.5G spectrum illumination. To obtain the optimum device performance, first, the ETL thickness has been varied from 10 to 100 nm, where it is found that above 20 nm, the device performance starts to diminish. Further keeping the ETL thickness at 20 nm, the absorber thickness has been tuned from 1 to 10 µm. The simulation results show an optimal device performance at 2 µm absorber thickness. Keeping the thickness of ETL and absorber layer at 20 nm and 2 µm, respectively, the HTL thickness has been changed from 1 to 10 nm. As the thickness increases, the device performance diminishes because of recombination of charge carriers in the HTL. Thus, the device exhibits optimal performance at layer thicknesses of ETL, absorber, and HTL at 20 nm, 2 µm, and 1 nm, respectively. The simulation results indicate that the proposed solar cell outperforms in terms of power conversion efficiency (around 28%) and fill factor (≈87%). In addition to the proposed solar cell, the excellent optoelectronic characteristics of the investigated monolayer Ge_2_Se_2_ make it a potential candidate for other photo devices. This study may also be helpful in the search for excelling materials regarding prospective applications in photo devices.

## Data Availability

All data that supports the findings of this study is available in the published article and/or the supporting information to this article.
